# Predicting RNA hyper-editing with a novel tool when unambiguous alignment is impossible

**DOI:** 10.1186/s12864-017-3898-9

**Published:** 2017-07-10

**Authors:** Wilson H. McKerrow, Yiannis A. Savva, Ali Rezaei, Robert A. Reenan, Charles E. Lawrence

**Affiliations:** 1 Division of Applied Mathematics, Brown University, Providence, 02912 RI USA; 2 Molecular Biology, Cell Biology and Biochemistry, Brown University, Providence, 02912 RI USA

**Keywords:** RNA editing, Hyper-editing, Alignment, Repetitive element, Transposable element, dsRNA

## Abstract

**Background:**

Repetitive elements are now known to have relevant cellular functions, including self-complementary sequences that form double stranded (ds) RNA. There are numerous pathways that determine the fate of endogenous dsRNA, and misregulation of endogenous dsRNA is a driver of autoimmune disease, particularly in the brain. Unfortunately, the alignment of high-throughput, short-read sequences to repeat elements poses a dilemma: Such sequences may align equally well to multiple genomic locations. In order to differentiate repeat elements, current alignment methods depend on sequence variation in the reference genome. Reads are discarded when no such variations are present. However, RNA hyper-editing, a possible fate for dsRNA, introduces enough variation to distinguish between repeats that are otherwise identical.

**Results:**

To take advantage of this variation, we developed a new algorithm, RepProfile, that simultaneously aligns reads and predicts novel variations. RepProfile accurately aligns hyper-edited reads that other methods discard. In particular we predict hyper-editing of *Drosophila melanogaster* repeat elements in vivo at levels previously described only in vitro, and provide validation by Sanger sequencing sixty-two individual cloned sequences. We find that hyper-editing is concentrated in genes involved in cell-cell communication at the synapse, including some that are associated with neurodegeneration. We also find that hyper-editing tends to occur in short runs.

**Conclusions:**

Previous studies of RNA hyper-editing discarded ambiguously aligned reads, ignoring hyper-editing in long, perfect dsRNA – the perfect substrate for hyper-editing. We provide a method that simulation and Sanger validation show accurately predicts such RNA editing, yielding a superior picture of hyper-editing.

**Electronic supplementary material:**

The online version of this article (doi:10.1186/s12864-017-3898-9) contains supplementary material, which is available to authorized users.

## Background

The advent of deep sequencing methodologies has opened up new opportunities to study non-coding RNA. Of particular interest are repetitive elements that form double-stranded (ds) RNA when transcribed. Long, perfect dsRNA stimulates innate immunity, regulates gene transcription, and has been implicated in a variety of neurological and autoimmune disorders [1, 2]. The fate of dsRNA depends on its interaction with RNA binding proteins. Possible fates include cleavage by dicer leading to gene silencing [3], suppression by TDP-43 related proteins [4], which have been been implicated in neurodegenerative disease [5], and hyper-editing by adenosine deaminase acting on RNA (ADAR) enzymes.

Recent evidence suggests that ADAR inhibits RNA interference [6] and the induction of innate immunity [7]. Both of these interactions seem to occur when ADAR competes with other enzymes for dsRNA substrates. The large number of I-U mispairs introduced by hyper-editing likely destabilizes those substrates, making them unavailable to other dsRNA enzymes [8]. Finding the genomic sources of hyper-edited dsRNA and describing the pattern of editing therein will improve our understanding of how ADAR functions within dsRNA pathways. It will also reveal highly expressed long, perfect dsRNA that may be important for other cellular pathways.

RNA editing by ADAR enzymes was first recognized for its exquisite specificity in modifying particular adenosine (A) residues to inosine (I) in structured double-stranded regions of pre-mRNAs. Because inosine is recognized as guanosine (G) by all cellular machines, including the ribosome, specific editing has the potential to change the amino acids encoded by mRNA. However ADAR enzymes have another activity on (nearly) perfect dsRNA: Hyper-editing can convert up to 50% of adenosines to inosine within the double-stranded region [9]. Hyper-editing of endogenous RNAs was first reported in human ALU elements, a class of transposable elements comprising about 10% of the human genome sequence and numbering over one million copies [10]. Analyses of hyper-editing revealed far more editing sites in repetitive elements than the known examples of specific editing in protein-encoding RNAs. As next-generation sequencing has become cheaper, new studies, using new sequencing methods and analyses, have increased the number of known editing sites and expanded our understanding of ADAR activity [11–15].

However, the ability of these studies to find hyper-editing in long, perfect dsRNA and to accurately estimate the level of editing at a given hyper-edited position is limited: They must discard reads that have no best alignment to a reference genome or risk widespread false positive predictions. By its nature, long dsRNA consists of a sequence followed by its reverse complement. This self-complementarity ensures that a read originating from the interior of such a molecule will align equally well on both the forward and reverse strand. To make matters worse, dsRNA is most likely to appear when repetitive elements are present, forming when two copies occur nearby but in opposite orientation or within certain self-complementary sequences. Thus, reads originating from within a long, perfect duplex are unlikely to have a single best alignment. Therefore, methods that discard reads with ambiguous alignment cannot provide a full picture of hyper-editing. Long read sequencing technologies do present a possible solution. However methods that can align short hyper-edited reads to dsRNA are needed, because short read data sets are cheap and ubiquitous.

To confront this challenge, we employed a probabilistic model that iteratively aligns reads and finds novel sequence changes, including hyper-editing and SNPs. While hyper-editing is the focus of this application, we also address other sequence modifications that may be confused with hyper-edits. For this purpose we used a three-component Dirichlet mixture model [16] to separate SNPs, hyper-edits, and positions that only differ from the reference by read error. We also estimate expression levels to further refine our alignment.

Here we present RepProfile, an algorithm that employs the expectation maximization (EM) algorithm [17] to find the read alignments, SNPs, hyper-editing patterns, and expression levels that are most likely under our model. This EM algorithm alternates between averaging over hidden variables (in this case, the alignment) in its E-step and estimating the hyper-editing, SNPs and expression (henceforth called the genome profile [18]) that maximize the likelihood of those averages in its M-step. While the alignment of a read to the reference genome may be ambiguous, as the algorithm refines its estimate of the genome profile, the probability of the correct alignment can grow to a point of near certainty if enough informative positions (nucleotides that distinguish between repeat copies) are identified, even when the genomic sequences of repetitive elements are identical. Because the expected alignment must be recalculated at each E-step, RepProfile is potentially computationally intensive. Thus, RepProfile is built to consider one repeat family at a time. A widespread analysis can be done by running RepProfile on many repeats in parallel.

Several methods have been proposed that consider read alignment and inference jointly, but none make use of novel sequence variation to improve alignment. TEtranscripts [19] uses the EM algorithm to learn expression levels in repetitive sequence. The algorithm of Wang et al. [20] is similar, but uses Gibbs sampling instead of EM and is designed for application to Chip-seq. The algorithm of Parks et al. [21] considers how genomic rearrangement affects read alignment. We are, as far as the authors know, the first apply such methods to position variation, including SNPs and hyper-editing.

## Results

RepProfile was used to predict hyper-editing in transposable elements from 2x100bp Illumina sequence reads from whole head *Drosophila melanogaster* RNA. RepProfile was run on each transposable element (TE) family in parallel. This included all repeats in the UCSC genome browser (genome.ucsc.edu) repeatmasker track except simple repeats, low complexity repeats, rRNA and satellites, a total of 29 megabases. Hyper-editing is not limited to TEs, but they are a common source of dsRNA, and RepProfile was designed to find hyper-editing in TEs. Repeat families that are a prefix of other families were merged. Thus, for example, PROTOP, PROTOP_A and PROTOP_B were considered together. Similarly the LTR and interior portions of RNA transposons were merged. RepProfile aligned 8.3 millions reads (totaling 1.66 gigabases) and predicted a total of 30,185 edit sites.

In this section we focus on predictions in FB4_DM, PROTOP and DNAREP1_DM. RepProfile predicts the most widespread hyper-editing in FB4_DM. Hyper-editing of PROTOP repeats was already described in [6], and DNAREP1_DM shows how imperfect helices can be hyper-edited. A note about each family with at least 1000 predictions can be found in the Additional file 1. A full list of all predictions can be found in Additional file 2: Table S3. We use simulation and clone validation to show the accuracy of RepProfile.

### Simulations support accuracy of RepProfile

RepProfile and competing methods were tested against three different simulations of hyper-editing. In the first reads are simulated from a hypothetical repeat family consisting of 24 identical copies of a random 1kb sequence: 20 isolated copies (10 in each orientation) and 2 oppositely oriented pairs that are simulated to be hyper-edited (one on each strand). In the second, we simulate reads from the FB4_DM repeat, including editing only at the sites observed in our clone data (see below). In the third simulation, FB4_DM repeats are chosen at random to be hyper-edited. In both FB4_DM simulations, reads are simulated in proportion to observed expression levels.

Reads drawn from the hypothetical repeat family show that RepProfile is able to provide accurate alignment to highly repetitive sequence. Because the genome sequence of these repeats are identical, no read that falls entirely within a repeat has a unique alignment to the hypothetical repeat reference. Nevertheless, RepProfile is able to align 90% of reads that fall entirely within one of the hyper-edited duplexes to the learned profile with mapping quality 30+ (estimated probability of misalignment ≤0.1*%*). 99.9% of these reads are aligned correctly, allowing RepProfile to predict editing sites with high sensitivity and PPV (Table 1).

**Table 1:** Sensitivity and PPV for RepProfile and competing methods in the three different simulations

Exact Rep	Sens.	PPV
RepProfile	0.94	0.99
Porath	0.07	1.0
EER uniq	0.36	0.99
EER +rep	0.90	0.14
Clone		
RepProfile	0.95	1.0
Porath	0.13	0.97
EER uniq	0.55	0.82
EER +rep	0.91	0.42
Random		
RepProfile	0.87	0.99
Porath	0.50	0.98
EER uniq	0.70	0.98
EER +rep	0.86	0.74

*Top*: Simulation from 24 identical copies of a hypothetical repeat. *Middle*: Simulation of editing predicted by clones. *Bottom*: Random hyper-editing of FB4_DM

**Fig. 1:**
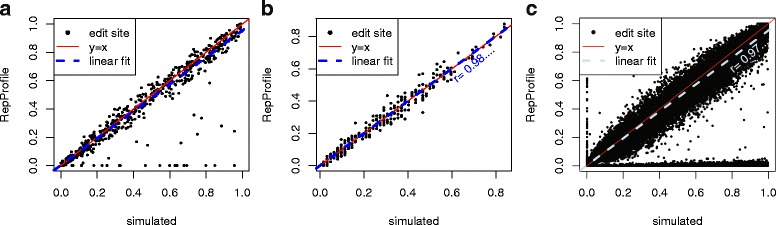
Agreement between simulated and predicted RNA-editing levels. Level of RNA-editing in simulation vs prediction for each position that is either simulated or predicted to be edited. **a** Edits simulated from hypothetical exact repeat copies. **b** Simulation using edits predicted by clone sequence. **c** Simulation of hyper-editing at random FB4_DM. Because this simulation reflects the varied coverage of an actual RNA-seq experiment, many simulated edit sites are covered by few of no reads (points along x axis)

In addition to RepProfile, we predicted edit sites using the method of Porath et al. [12] and by finding editing enriched regions (EERS) [15], either using all reads (+rep) or only using reads for which at least one end aligns uniquely (uniq). Neither of these methods uses a similar strategy to RepProfile. In particular, the Porath et al. method considers only reads with a large number of A to G changes. However there are few published methods showing success predicting RNA-editing in repeats, and the comparison shows that hyper-editing of long, perfect dsRNA cannot be found using a simpler method. Table 1 shows sensitivity and positive predictive value (PPV) for each method applied to each simulation. RepProfile provides the highest sensitivity in all three simulations, while maintaining a PPV of 99% or above. RepProfile also provides accurate estimation of the editing level across all simulations (see Fig. 1).

In the clone simulation, editing occurs at A/G informative positions. Thus hyper-edited reads can align uniquely but incorrectly, explaining the diminished PPV when finding EERs with unique reads. RepProfile’s diminished sensitivity in the random hyper-editing simulation is due to the fact that many simulated edit sites occur in low coverage regions. As repeats accumulate mutations, unique alignments become possible, but the repeats also tend to lose their dsRNA structure. Because edit sites are not limited to dsRNA in the random hyper-editing simulation, more hyper-edited reads align uniquely, allowing the competing methods to perform better.

### RepProfile predictions are validated by Sanger clones

A hyper-edited FB4_DM in the gene retinal degeneration A (rdgA) was chosen for validation. Not only is this repeat not unique, it is also internally repetitive, making alignment particularly challenging. rdgA is expressed almost exclusively in the nervous system [22], as is dADAR protein. 62 sequences were generated by cloning RT-PCR amplicons from the sequence spanning chrX:8,928,544-8,929,835 in the dm6 genome assembly (available from the UCSC genome browser: genome.ucsc.edu). The cloned sequences showed a small deletion spanning chrX:8,928,786-8,929,278 and so the FB4_DM reference was updated to include this deletion.

**Fig. 2:**
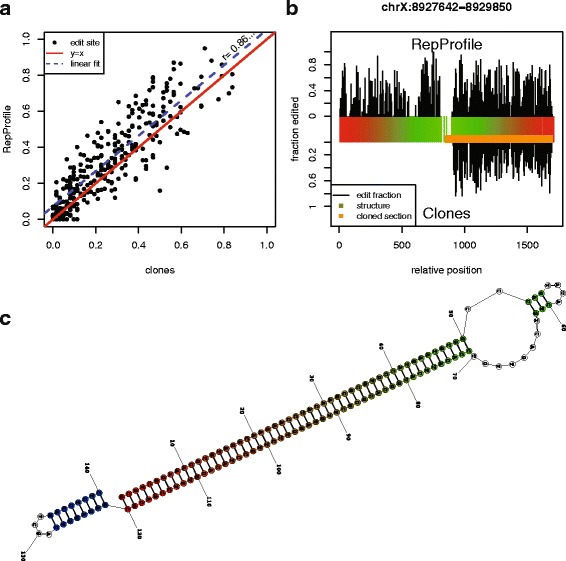
Agreement between RepProfile and clone validation. **a** Fraction of editing predicted by clones vs fraction predicted by RepProfile. RepProfile and clones show good agreement in the rdgA FB4_DM element. **b** RNA-editing predictions aligned to RNA secondary structure as predicted by RNAstructure [39, 40]. Positions are colored so that aligned pairs are the same color. Unpaired positions are not colored. Both methods predict hyper-editing only in the helical portion of this repeat, consistent with the fact that ADAR is a dsRNA binding protein. **c** Explanation of RNA structure coloring. A gradient from *red* to *green* to *blue* is stretched across the length of the sequence. Paired positions are colored according to the minimum of their position and the position they are paired to. Unpaired position are not colored

The Sanger sequences confirm the pattern of hyper-editing predicted at this locus, with each clone displaying a distinct pattern of edited sites. Of 322 adenosines in the clone region, editing is observed at 280 positions (87%) in at least 1 clone. Each clone is edited at an average of 76.7 adenosines (24%). RepProfile predicts editing at 269 of the 280 positions edited in the clone sequences (sensitivity = 96%). RepProfile predicts editing at an additional 11 sites, yielding a PPV of 96%. As Fig. 2 a shows, RepProfile accurately predicts editing levels. Among positions that show evidence for editing both in RepProfile and in the clones, RepProfile overestimates editing by 6%, with a standard deviation of 11%. Figure 2 b provides a site-by-site comparison of predicted and validated editing.

Using reads for which at least one end aligns uniquely, the method of EERs [15] predicts only 31% of the cloned edit sites. All predictions made by the EER method are supported by the clone validation. Applying the method of Porath et al. [12] to our data yields 17 editing sites in this FB4_DM element, but fails to find any editing in the cloned region. These sensitivity estimates are lower than those estimated in simulation, indicating that there are additional alignment challenges not included in our simulation. Similarly our Helicos single-molecule sequencing (SMS) results [13] find 2 tier 1 and 10 tier 2 edit sites in this element, but none in the cloned region. Rodriguez et al. [11] and Ramaswami et al. [14] fail to predict any editing in this FB4_DM. The lack of FB4_DM hyper-editing in these published lists is unsurprising as they all rely on unambiguous alignment of short reads.

### FB4_DM repeats are highly hyper-edited

The FB4_DM sequence is almost entirely a perfect inverted repeat, which has the capacity, if transcribed, to fold back and form long, (nearly) perfect dsRNA (see Additional file 3). Thus, transcripts containing FB4_DM in pre-mRNA are potentially excellent ADAR hyper-editing substrates. Indeed, RepProfile predicts frequent hyper-editing of FB4_DM elements.

**Fig. 3:**
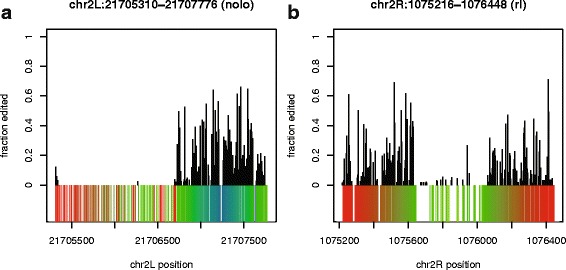
RepProfile predictions for two FB4_DM. Alignment of RepProfile predictions to RNAstructure [39, 40] secondary structure predictions. Secondary structure is colored as in Fig. 2b/c. **a** An FB4_DM element in the gene nolo. **b** An FB4_DM element in the gene rolled (rl)

In addition to the element in rdgA described above, there are four other FB4_DM that are predicted to be hyper-edited by RepProfile with highest confidence (see discussion). These predictions appear in the genes no-long-nerve-cord (nolo), Pur-alpha, Maf1 and rolled (rl). Across these five repeats (including rdgA), 1681 editing sites are predicted. Interestingly, like rdgA, these genes are known to be involved in proper cell-cell communication in the nervous system, particularly in the correct function of synapses [23–26]. Two (Pur-alpha [24] and rdgA [22]) are associated with neurodegneration. Seven more FB4_DM (see Table 2) are predicted to be edited by RepProfile at slightly lower confidence (see Discussion). This brings the total number of predicted edit sites to 4384. Five of these seven are also in genes that have been shown (or are predicted) to play roles in proper neuronal maintenance and function [27–31]. Two examples of FB4_DM hyper-editing are shown in Fig. 3.

**Table 2:** FB4_DM that are predicted to be hyper-edited. Predictions with a yes in the last column are RepProfile’s most confident predictions

Position	Gene	Most confident
chr2L:21705310–21707776	nolo	Yes
chr2R:1075216–1076448	rl	Yes
chr2R:1521733–1522952	Maf1	Yes
chr4:560886–562920	Pur-alpha	Yes
chrX:8927642–8929850	rdgA	Yes
chr3L:4361048–4362834	Cip4	No
chr3L:8019759–8024110	nmo	No
chr3R:1971337–1972661	Myo81F	No
chr3R:21609064–21611636	inR	No
chr3R:22450601–22453179	CG34376	No
chrX:11645168–11648276	Ptp10D	No
chrX:2132105–2133551	ph-p	No

If there is a No in the last column, RepProfile was able to align reads without predicting hyper-editing at this repeat, but not predicting hyper-editing at these repeats yielded a lower posterior probability

There are seven additional genes containing FB4_DM elements that are predicted to form dsRNA, but are not predicted to be edited: CG11873, CG17600, CG42238, kek5, kirre, Pka-R1, vtd. Only two (Pka-R1 and vtd) of these genes are annotated with neuron-related GO terms in flybase [32] (as of January 1, 2017). It is possible that while these genes are edited in neurons, the edited reads are overwhelmed by transcription in cells that do not express ADAR. Alternatively, these RNA duplexes may be targeted by another dsRNA binding protein, making them unavailable to ADAR.

### DNAREP1_DM repeats form imperfect helices that are partially edited

**Fig. 4:**
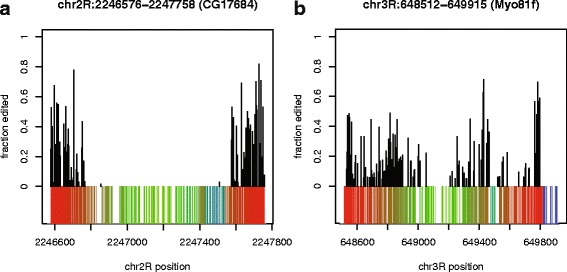
RepProfile predictions for two DNAREP1_DM. Alignment of RepProfile predictions to RNAstructure [39, 40] secondary structure predictions. Secondary structure is colored as in Fig. 2b/c. **a** A complementary pair of DNAREP1_DM elements in the gene CG17684. **b** A complementary pair of DNAREP1_DM elements in the gene Myo-81f

While shorter (up to 500 nt) the 5,802 DNAREP1_DM elements in the fly genome play an analogous role to that of ALU repeat elements in the human genome. As with ALU repeats, it is not uncommon for two DNAREP1_DM elements to be oriented in opposite directions in the same gene, or even to be opposite and adjacent. However DNAREP1_DM instances tend to be quite divergent, and so these DNAREP1_DM form imperfect helices that are edited to a lesser extent than FB4_DM. Figure 4 shows the hyper-editing and structural predictions for two pairs of DNAREP1_DM that are adjacent and opposite in orientation. Table 3 lists all the DNAREP1_DM that are predicted to be hyper-edited. RepProfile predicts 685 edited positions in DNAREP1_DM. Many of these genes are also relevant to the nervous system [33–36].

**Table 3:** DNAREP1_DM that are predicted to be hyper-edited

Position	Gene	dsRNA
chr2L:22996546–22999081	Intergenic	Adjacent +/-
chr2R:1305412–1314667	Intergenic	Unknown
chr2R:2245576–2248758	CG17684	Adjacent +/-
chr2R:3107642–3109943	dpr21	Same gene +/-
chr2R:4900040–4902165	CG44102	Same gene +/-
chr2R:6823192–6825246	Intergenic	Unknown
chr3L:23034337–23037599	nrm	Adjacent +/-
chr3L:24175963–24178195	Snap25	Same gene +/-
chr3L:24183640–24186213	Snap25	Same gene +/-
chr3L:24224373–24226438	snap25	Same gene +/-
chr3L:25653710–25655922	CG45782	Same gene +/-
chr3R:1453181–1455383	Myo81F	Same gene +/-
chr3R:1593187–1595257	Myo81F	Same gene +/-
chr3R:1627526–1629596	Myo81F	Same gene +/-
chr3R:647512–650915	Myo81F	Adjacent +/-
chr3R:888752–890953	Myo81F	Same gene +/-
chr4:1147092–1149608	CG32017	Adjacent +/-
chr4:859109–861284	CG11148	Same gene +/-
chrX:142931–144983	tyn	Same gene +/-

Adjacent +/- indicates that there is a DNAREP1_DM within 2kb that is in the opposite orientation. Same gene +/- indicates that there is a DNAREP1_DM in the same gene that is in the opposite orientation. Unknown means that neither of these two conditions apply

### Alignments to PROTOP show hyper-editing of the previously described Hoppel killer element

The most probable solution found by RepProfile includes 38 hyper-edited PROTOP, PROTOP_A and PROTOP_B elements containing a total of 4326 edit sites. Here we focus on the five most confident predictions (see Discussion). All five are pairs of PROTOP(A/B) that are adjacent but opposite in orientation (see Table 4). These repeats contain a total of 973 predicted edit sites, 697 of which are in the Hoppel killer (Hok) element [6].

**Table 4:** PROTOP(A/B) that are predicted to be hyper-edited at the highest confidence

Position	Gene	dsRNA
chr3L:28002423–28003664	CG17514	Adjacent +/-
chr3R:2420941–2423648	Myo81F	Adjacent +/-
chr3R:3788494–3789401	Intergenic	Adjacent +/-
chr4:1257060–1260307	cadps	Adjacent +/-
chr3L:24327899–24328803	nvd	Adjacent +/-

Adjacent +/- indicates that there is a PROTOP(A/B) within 2kb that is in the opposite orientation. Same gene +/- indicates that there is a PROTOP(A/B) in the same gene that is in the opposite orientation. Unknown means that neither of these two conditions apply

**Fig. 5:**
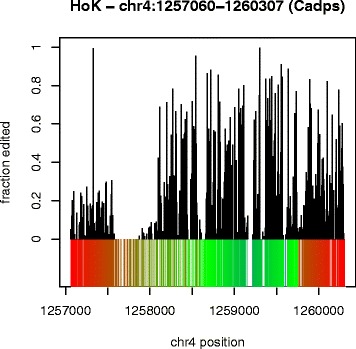
RepProfile predictions for the PROTOP_A elements that form Hok. Alignment of RepProfile predictions for Hok to the RNAstructure [39, 40] secondary structure prediction. Secondary structure is colored as in Fig. 2 b/c

Our previous work [6] demonstrated that dADAR proteins, as well as other dsRNA-binding proteins, localize to the Hok element in vivo, but using SMS we only found a small number of editing sites in Hok [13]. However with RepProfile we are able to predict drastically more editing – a result that is more in line with the strong evidence of ADAR activity at this locus. Figure 5 shows predicted editing for Hok. Hok contains three highly similar PROTOP_A elements, so there may be structural conformations other than the one illustrated in Fig. 5.

### ADAR edits in short runs

**Fig. 6:**
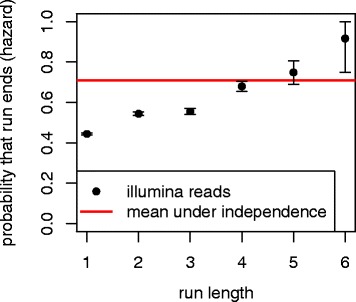
Estimated hazard function for length of editing run. The estimated probability that a run of FB4_DM editing will end after a given number of edited A’s, with 95% binomial confidence intervals. The red line is the marginal probability that an A following an A at helix depth at least 25 will not be edited in a particular read. The estimates indicate that as a run of edits gets longer, it is more likely to end

FB4_DM sequences contain long strings of consecutive adenosines, sometimes more than ten adenosines long. We analyzed the hyper-editing of consecutive adenosines on a read-by-read basis to understand how ADAR edits long, perfect dsRNA. When analyzing runs of edited adenosines that are followed by another base, we do not not know whether the run would have continued had there been more adenosines to edit. Thus we have (a discrete version of) the lifespan estimation from censored data problem analyzed by Kaplan and Meyer [37]. Runs of edited adenosines that are followed by a base that is not adenosine are considered to be censored, as the run may have continued were there more adenosines to edit. The hazard function,

$${} \begin{aligned} P(\text{run}\,\,\text{length}&=n|\text{run}\,\,\text{length} \geq n)\\ &\approx \frac{\#n\,\mathrm{long,}\,\text{uncensored}}{(\#n\,\mathrm{long,}\,\text{uncensored})+(\#>n\,\text{long})} \end{aligned} $$

is shown in Fig. 6. Short runs are less likely to end than would be predicted from context alone. However as the run gets longer the probability that the run will end increases. This is consistent with the theory that as ADAR edits, it disrupts the dsRNA structure, introducing I-U mispairs and making future editing less likely.

### Short helices are rarely edited; the longest helices are edited most

To measure the affect of dsRNA structure on editing, we measure the length of helices in the five most confident FB4_DM hyper-editing predictions (Table 2) and the five adjacent +/- DNAREP1_DM repeats that are predicted to be hyper-edited (Table 3). Helices are allowed to include bulges of up to two bases on one or both sides of the helix, as small bulges have been shown not to interrupt hyper-editing [38]. Structure predictions are by RNAstructure [39, 40].

**Fig. 7:**
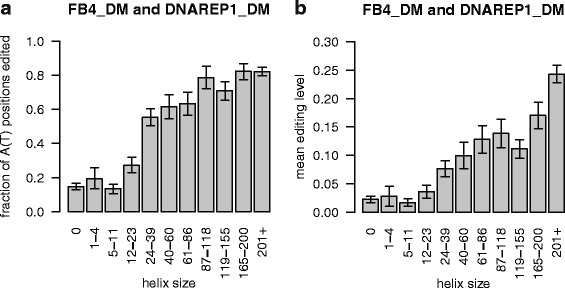
Probability that editing will be predicted as a function of helix size. **a** The fraction of A positions at which any amount of editing is predicted, binned according the helix size, allowing bulges of of up to two bases for the five most confident FB4_DM hyper-editing predictions (Table 2) and the five adjacent +/- DNAREP1_DM repeats that are predicted to be hyper-edited (Table 3). Error bars are 95% binomial confidence intervals. **b** Mean fraction of editing, binned according the helix size as in part A. Error bars are 95% normal approximation confidence intervals. Note that in both A and B, larger bins are supported by many positions but only a few helices, so confidence intervals may be overly tight. Structure predictions by RNAstructure [39, 40]

We find that editing is rare in short helices and that it is most frequent in long helices. RepProfile predicts editing at only 13% of adenosines in helices that are fewer than 18 basepairs long, but at 80% of adenosines in helices that are longer than 64 basepairs. This is consistent with evidence that ADAR does not bind to helices shorter than 15–20 basepairs and is most efficient when editing helices longer than 100 basepairs [9]. Figure 7 shows editing binned by helix size.

In the 819 basepair rdgA FB4_DM helix (the longest in this analysis), 84.1*%* of adenosine positions are predicted to be edited, but in individual transcripts only 27.7*%* of adenosines are edited on average. This editing of 27.7*%* of adenosines is less than the 50−60*%* seen in vitro [9]. This difference could be because the editing reaction is not allowed to complete in vivo, because transcripts are sequenced before they are fully edited, or because some copies are sequenced from cells with low levels of ADAR.

### The nucleotide context of our predictions reflects known ADAR preferences

**Fig. 8:**
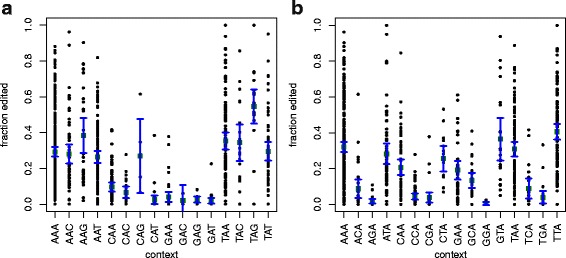
Fraction of editing at predicted edit sites as a function of three-letter context. **a** The three-letter context centered at the edited base. The 5’ base is much more predictive of editing level than the 3’ base. **b** The three-letter context ending at the edited base. The base two back from an edited position has only a small effect

We investigated the effect of preceding and following bases on the fraction of editing at an adenosine (A) position. We consider only positions that are in the five most confident FB4_DM predictions and are also at least 25 bases away from the nearest unpaired position – a total of 865 adenosines. Figure 8 shows the fraction of editing for sites in each three-base context.

The preceding base has a strong effect on the fraction of editing. Predicted edit sites following T are edited in the highest fraction of reads (mean = 0.35). Sites following A are slightly less likely to be edited (mean = 0.29). Sites following C are much less likely to be edited (mean = 0.08) and sites following G are rarely edited (mean = 0.03.) Each pairwise comparison has t-test BHY [41] FDR less than 0.003. Consistent with evidence that ADAR edits in runs, this 5’ preference affects the following base: Adenosines preceded by AA or TA are edited more often than adenosines preceded by CA or GA (pairwise FDRs all less than 0.015.)

The following base has a smaller effect on the fraction of editing. Adenosines followed by G are edited most often (mean=0.35), followed by A (mean=0.26), C (mean = 0.24) and T (mean = 0.21.) However the only statistically significant result is that adenosines followed by G are more likely to be edited (*p* value = 0.0018.) Our results for 3’ and 5’ base preferences agree with those found in previous studies [42–45].

### Run time per RepProfile step scales with the number of candidate alignments; the number of steps depends on the amount of editing

**Fig. 9:**
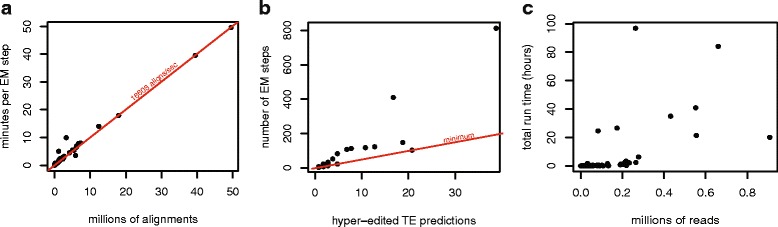
EM step run time. EM run time on 8 cores. **a** The RepProfile bottleneck occurs during the E step where about 16,608 candidate alignments are processed per second. **b** The red line indicate the minimum number of steps run by RepProfile. More steps are run if many local maxima are found. **c** RepProfile runs time vary widely

For most repeats, calculating the probability of each candidate alignment in each E step forms a bottleneck. Thus the time to complete a single EM step scales with the number of candidate alignments (Fig. 9a). This makes run time difficult to predict a priori as the number of candidate alignments depends not only on the number of reads, but also on the number of candidate alignments per read. For example, there are five times as many reads that align to DNAREP1_DM repeats as there are reads that align to FW_DM repeats. However predicting hyper-editing in DNAREP1_DM takes less than half as long per step, because FW_DM repeats are much more similar to one another than are DNAREP1_DM repeats. Alignment probabilities are calculated independently, making the E step highly parallelizable. For FB4_DM, a single EM step takes 1353, 664, 365, 215, and 131 seconds on 1, 2, 4, 8, and 16 cores, respectively.

While EM usually converges in a small number of steps, as it runs, RepProfile suggests new initial conditions to explore alternate hyper-editing solutions. At minimum one initial condition is tried for each hyper-edited repeat (Fig. 9 b). However RepProfile will continue trying new initial conditions if a more likely solution is found with a different set of hyper-edited repeats. Most TEs (about 80%) run in 30 minutes or less on 8 cores, but six TEs required run times of a day or more (Additional file 2: Table S2, Fig. 9 c). The most computationally intensive repeat, PROTOP(A/B), required about 4 days of run time with 815 steps. Running RepProfile on all TEs required a total of 16 node-days at 8 cores per node.

### Summary

RepProfile provides accurate RNA hyper-editing predictions that are validated both by simulated data and by individual sequence clones. Our analysis reveals hyper-editing that is not – indeed we argue cannot be – found by other methods. In particular, we highlight the hyper-editing of long, perfect dsRNA formed by FB4_DM elements – a repeat whose relevance to hyper-editing was not previously known – in the introns of genes with synaptic function. We also estimate the level of editing – something that other methods do not do. In addition to finding many hyper-editing events in long, perfect dsRNA, our results show that ADAR often edits a run of adjacent adenosines; that editing is rare in helices less than 20 base pairs long but becomes more frequent as helix length increases; and that, consistent with previous findings, adenosines are more likely to be edited if they follow A or T than if they follow C or G.

## Discussion

The successes of RepProfile, both in simulation and validation, show that short reads can predict RNA editing even when standard alignment techniques cannot produce confident alignments. Even if repeats are locally identical, they are likely to form different RNA secondary structures in the context of different transcripts, leading to unique editing patterns. Additionally there may be cell-specific factors that further differentiate hyper-editing patterns. Thus, when endogenous dsRNAs are “marked" by ADAR modification with a unique editing pattern, RepProfile can distinguish between identical repeats.

As far as the authors know, RepProfile is the only tool capable of using RNAseq data to accurately find RNA hyper-editing (or position variation in general) within sequences that form long, perfect dsRNA. RepProfile reveals RNA duplexes with hundreds of edited positions, where other methods, reliant on unambiguous alignment to single reference genome, find few or No sites. Because almost all RNAseq analysis methods rely on unambiguous alignment to a reference genome, it is likely that many studies have missed valuable insights regarding dsRNA. This is especially important for RNA hyper-editing as hyper-editing only occurs in dsRNA. While previous studies have been able to describe hyper-editing events, their descriptions are limited to dsRNA molecules that contain sufficient imperfections (bulges) for unambiguous alignment.

The major challenge for EM applications, such as RepProfile, is that EM is only guaranteed to find a local maximum, which may or may not be the global maximum. Thus, EM must be run with a variety of initial conditions in order to be confident that the global max has indeed been found. When applying RepProfile to real RNAseq reads, we often find several maxima, leading to the distinction between highly confident and regular hyper-editing predictions. The highly confident predictions are repeats that are predicted to be hyper-edited in all maxima. Highly confident predictions tend to occur when RepProfile can align paired reads such that one end is aligned to the hyper-edited repeat and the other is aligned outside the repeat. For FB4_DM, coverage of the flanking sequence is 13 times higher for highly confident predictions (vs 4 times higher for the repeat itself). As the flanking sequence tends to be more unique than the repeat itself, it is difficult to be sure which repeat is hyper-edited without these flanking reads. The failure to align reads outside the repeat could be due to repetitiveness in the flanking sequence, inaccuracies in the reference, or simply because of low coverage.

While repeatedly realigning reads allows for accurate predictions of hyper-editing in repetitive elements, it is time consuming. Thus RepProfile only considers one repeat family at a time. As each repeat can be considered in parallel, it is possible to use RepProfile to predict *Drosophila melanogaster* hyper-editing genome-wide. Even so, for prevalent, highly repetitive repeats such as PROTOP(A/B), RepProfile can take days to run. In a larger genome, such as the human genome, there may be repeats that are even more computationally challenging. Thus improvements may be necessary when applying RepProfile to a large genome. Such improvements could be made by creating new c extensions or by selectively updating alignment probabilities. RepProfile is written entirely in Python (with heavy computation done by numpy) and does not check whether the profile has changed significantly before recalculating alignment probability at each step.

Our analysis provides insight into how ADAR edits at the molecular level. We find that ADAR is more likely to edit adjacent adenosines, but is less likely to extend long runs of editing. This indicates that ADAR edits processively, but that as it edits it destabilizes the helix, causing the enzyme to detach. The finding that short bulges do not interrupt RNA-editing [38] explains why ADAR activity does not slow until the editing run lengthens. Our data confirms the results of in vitro experiments showing that ADAR does not readily bind to short helices and that long dsRNA is required for maximum editing efficiency [9]. We also confirm the highly replicated result that ADAR has a strong 5’ preference for A or U over G or C, but weak 3’ preferences [42–45]. Good agreement with these results provides further evidence that RepProfile gives an accurate picture of RNA hyper-editing.

The predictions made by RepProfile point to the possibility that hyper-edited TEs play a functional rule – a question that deserves further investigation. Most of our predictions, indeed all of our most confident FB4_DM predictions, are in highly-conserved genes with synaptic functions in both invertebrates and vertebrates. This similarity of function is not likely to arise by random transposable element (TE) insertion, providing evidence for the domesticated use of TEs to regulate neuronal gene expression. Of course this is merely an observed correlation and it possible that some property of neuronal genes coincidentally facilitates hyper-editing of TEs. Our results provide a baseline picture of hyper-editing in these genes. These sites can now serve as targets for future studies investigating how hyper-editing is controlled and how it affects gene regulation.

While we have used RepProfile to predict hyper-editing, it also uses SNPs and expression levels to differentiate between repeats. In addition to the hyper-editing application described here, we envision that RepProfile is capable of finding unreported SNPs in repetitive sequence and of reconstructing the sequences of novel transposable element insertions. Indeed any sequencing experiment relies on an accurate alignment, and our results show that RepProfile can provide high quality alignment to repeats. The need for higher quality alignments may be especially great in differential expression experiments where failing to account for variation can lead to biased results [46]. Thus, RepProfile has the potential to improve a wide range of RNAseq experiments.

## Conclusion

It is often not possible to unambiguously align a single read, considered in isolation, to a repetitive reference genome. As a result, most analysis pipelines only consider unique regions of the genome, failing to provide any results about long, perfect dsRNA. Not only is such dsRNA the prime target for ADAR, proper regulation of dsRNA, in which ADAR plays a crucial role, is necessary for normal neuronal function. RepProfile provides accurate hyper-editing predictions in dsRNA, showing that, in the case of RNA editing at least, unambiguous alignment (to a reference genome) is not necessary for accurate inference. By building a complete probabilistic model that not only considers the information that aligned reads provide about hyper-editing, but also the information that hyper-editing provides about those read alignments, we are able to provide a more complete and more accurate picture of how ADAR edits endogenous dsRNA. We find that ADAR edits in short runs, and we observe the most hyper-editing in FB4_DM repeats that are in the introns of genes with synaptic functions, two of which are associated with neurogeneration, hinting at a regulatory role for hyper-editing. Previous studies of RNA editing in *Drosophila melanogaster* have failed to identify hyper-editing in this repeat, showing that a method, such as RepProfile, that accurately aligns short reads to dsRNA is necessary to begin teasing apart dsRNA pathways, and to understand the regulatory role of ADAR.

## Methods

### RepProfile Algorithm

Glossary of Random Variables:

- *R*=*R*_1_,…,*R*_*m*_ is the set of *m* Read sequences.
- *A*=*A*_1_,…,*A*_*m*_ is the Alignment of each read.
- *X*=*X*_1_,…,*X*_*r*_ is the relative eXpression of each repeat, where *r* is the total number of repeats.
- *G*=*G*_1_,…,*G*_*n*_ is the Genome profile (probability of A/C/G/T sequenced) at each position in each repeat, where *n* is the total number of positions.
- *H*=*H*_1_,…,*H*_*r*_ are the Hyper parameters, representing underlying sources of variation in G (hyper-editing in our applictation).
- *T*=*T*_1_,…,*T*_*n*_ are the variation Types of each position such as SNPs or edited positions.
- *U*(*A,R*)=*U*_1_,…,*U*_*n*_ are the number of A, C, G, T aligned at each position.
- *V*(*A,R*)=*V*_1_,…,*V*_*r*_ are the number of reads aligned to each repeat.

**Fig. 10:**
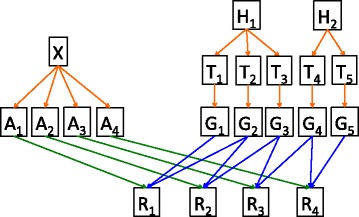
Bayesian network for RepProfile probability distribution. Directed graph describing the probability distribution used by RepProfile as a Bayesian network. Reads, *R*, depend on the profile, *G*, and the alignment, *A*. The profile depends on the position types, *T* (e.g. edited position), which depend on the repeat types *H* (e.g. hyper-edited repeat.) The alignment *A* depends on expression levels, *X*

The hierarchy in Fig. 10 generates a probability distribution across read sequences. Repeat expression levels, modeled on the left side, combine with nucleotide variations, modeled on the right, to generate read sequences. We can use EM to maximize the joint probability, which as the two are proportional, also maximizes the posterior distribution conditioned on the observed read sequences. To streamline the computation, only potential alignments suggested by a standard aligner are considered.

**Fig. 11:**
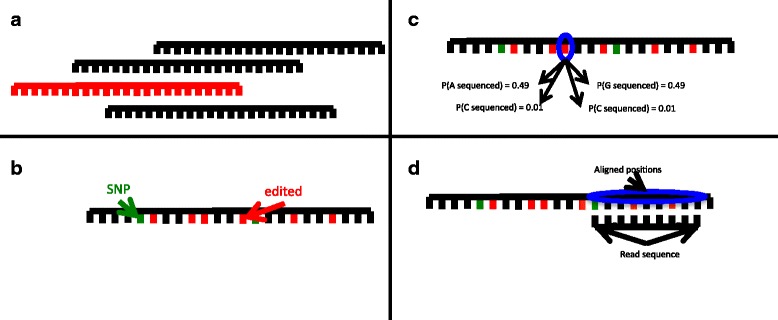
Cartoon description of RepProfile probability distribution. **a** There are many repeat copies in the genome. Each has some probability of being hyper-edited. *H* tells us which are hyper-edited and which are not. **b** Within each repeat, each position has a chance of being a SNP and each A in a hyper-edited repeat has a chance of being edited. *T* indicates which positions are SNPs and which are edited. **c** Any base can be sequenced at any position. *G* gives the distribution over A/C/G/T at each position. **d** Each repeat has a probability of generating the next read (defined by *X*), and *A* is the specific aligned position for the read. The read bases, *R*, are drawn from the corresponding distributions in *G*

The model of nucleotide changes begins with a repeat genome consisting of all copies (repeat elements) of a particular repeat in an organism’s reference genome. Each repeat element, *k*, is in one of several states, *H*_*k*_. In our analysis of hyper-editing, the states are: hyper-edited on the forward strand, hyper-edited on the reverse strand and not hyper-edited (Fig. 11 a). Similarly, each genomic position, *i*, within each repeat is in one of several states, *T*_*i*_. In our hyper-editing model, the states are: edited, SNP and neither (Fig. 11 b). The probability of a particular position being in a particular state depends on the repeat state, *H*_*k*(*i*)_. For instance an adenosine can only be in the edit state, if it is in a repeat that is hyper-edited on the forward strand. Conditioned on the state, *T*_*i*_, we model *G*_*i*_(*x*), the probability of nucleotide *x*, at position *i*, using a Dirichlet distribution – a distribution of distributions over the four-letter nucleotide alphabet {A,C,G,T } (Fig. 11 c). Thus, *G*_*i*_(*x*) is sampled from a mixture of Dirichlets, with *T*_*i*_ being the mixture component. See the Additional file 4 for the exact parameter values that define *P*(*H,T,G*).

In parallel, a distribution across genomic positions, *X*, defines the probability of sequencing a read that starts at a particular position. *X* is assumed to be constant across positions in a single repeat element. Thus, *X* can be thought of as defining the relative expression level of each repeat element. Alignments, *A*_1_,*A*_2_,…,*A*_*m*_, are drawn from *X* (Fig. 11 d). Insertions and deletions are inserted according to an affine gap probability. Given a set of aligned positions, *A*_*j*_, and distributions over nucleotides at those positions, $G_{A_{j}}$, the probability of a read sequence, *R*_*j*_, is the product across read letters of the probability of those read letters: $\prod G_{A_{j}}(R_{j})$. The assumption here is that, conditioned on the profile, the read sequence at each position is independent. This assumption is contradicted by our own observation that ADAR edits in short runs. However assuming this independence is necessary for efficient computation, and our simulation and validation shows that this assumption does not prevent accurate estimation. Distinguishing repeats that are hyper-edited from those that are not (*H*) allows us to preserve the key dependence: that edit sites tend to localize.

After conducting an RNAseq experiment, we observe a read set *R*. If we assume that *R* is drawn from the distribution described above then we can use EM to estimate *X,H,T* and *G* by maximizing *P*(*X,H,T,G,R*)∝*P*(*X,H,T,G*|*R*), and treating the alignment, *A*, as a hidden variable.

If we reparameterize *A* and *R* to new random variables: *U* that counts the number of A/C/G/T aligned each position and *V* that counts the number of reads aligned to each repeat, *P*(*A,R*|*G,X*) becomes an exponential family distribution:

$$\begin{array}{*{20}l}{} P(A,R|G,X) &\propto h(A) \prod_{k} X_{k}^{V_{k}(A)} \prod_{i=1}^{n}\prod_{x \in \{a,c,g,t\}}G_{i}(x)^{U_{i}^{x}(A,R) }\\ &= h(A)\exp [(U,V)\cdot(\log G, \log X)] \end{array} $$

where $U_{i}^{x}(A,R)$ is the number of nucleotide *x* aligned to position *i*, *V*_*k*_(*A*) is the number of reads aligned to repeat *k* and *h*(*A*) are indel probabilities. To perform an EM update, we need to calculate the following quantity:

$${} \begin{aligned} &G^{(t+1)},T^{(t+1)},H^{(t+1)},X^{(t+1)}\\ &\quad\quad=\underset{G,T,H,X}{\arg\max}{\mathbb{E}}\,[\!\log P(A,X,H,T,G,R)|R,G^{(t)},H^{(t)},X^{(t)} ] \end{aligned} $$

As Dirichlet distributions are conjugate to the exponential family above, the maximization can be completed as follows. First we consider terms depending on *X*:

$$\underset{X:\sum X=1}{\arg\max}(\alpha_{X}-1+{\mathbb{E}}_{A} [\!V])\cdot \log X=\frac{\alpha_{X}-1+{\mathbb{E}}_{A} [\!V]}{\sum \alpha_{X}-1+{\mathbb{E}}_{A} [\!V]} $$

where *α*_*X*_ are the Dirichlet parameters and ${\mathbb {E}}_{A}$ is expectation over A conditioned on (*R,G* ^(*t*)^,*H* ^(*t*)^,*X* ^(*t*)^).

Next, for fixed *T* we can maximize over *G*:

$$\begin{aligned} \underset{G:\sum G_{i} =1}{\arg\max}(&\alpha_{T}-1+{\mathbb{E}}_{A} [\!U])\cdot \log G\\ &=\frac{\alpha_{T}-1+{\mathbb{E}}_{A} [\!U]}{\sum\alpha_{T}-1+{\mathbb{E}}_{A} [\!U]}=\hat{G}_{T} \end{aligned} $$

Then we can maximize *T* for a given *H*:

$$\underset{T}{\arg\max} \log P(T|H)- \log Z_{T} +(\alpha_{T}-1+{\mathbb{E}}_{A} [\!U])\cdot \log \hat{G}_{T} $$

where *Z*_*T*_ is the Dirichlet normalization constant for *f*(*G*|*T*). Finally, we can find the optimal value of *H* for each repeat and then work backwards to get *T* and *G*. More details can be found in the Additional file 4.

It remains to calculate ${\mathbb {E}}_{A} [\!U]$ and ${\mathbb {E}}_{A} [\!V]$, which is done by summing the counts for each individual alignment:

$$\begin{aligned} {\mathbb{E}}_{A} [\!U]&=\sum_{j} {\mathbb{E}}_{A_{j}} [\!U(A_{j})]\\ &=\sum_{j}\sum_{A_{j}}P\left(A_{j} |R_{j},G^{(t)},X^{(t)}\right)U(A_{j}) \end{aligned} $$

$$\begin{aligned} {\mathbb{E}}_{A} [\!V]&=\sum_{j} {\mathbb{E}}_{A_{j}} [\!V(A_{j})]\\&=\sum_{j}\sum_{A_{j}}P\left(A_{j} |R_{j},G^{(t)},X^{(t)}\right)V(A_{j}) \end{aligned} $$

However it is not computationally feasible to sum over all possible alignments *A*_*j*_ for all reads *j* by brute force. In Hidden Markov Models, the forward and backward sum algorithm is usually used to achieve computational feasibility. However it is still *O*(*mnq*) for a repeat genome of length *n* and a dataset of *m* reads of length *q*. Thus it is still not feasible when reads number in the hundreds of millions. Fortunately for most bases at most positions, the probability of that base at that position will be small. Thus most of the possible alignments will have probability near 0. We can approximate the sum over alignments by considering only a small number of candidate alignments. In the case of hyper-editing, we allow candidate reads to have any number of A to G mismatches but only four other mismatches.

While each step of EM is guaranteed to produce a larger value of *P*(*G* ^(*t*)^,*H* ^(*t*)^,*T* ^(*t*)^,*X* ^(*t*)^|*R*), the process is not guaranteed to converge to the global maximum. In some cases, EM gets stuck at a local maximum. In many applications, EM is run many times with many different initial conditions. The solution that gives the largest value of *P*(*R,G,H,T,X*) is taken. As RepProfile runs, it creates new initial conditions by removing hyper-editing from each repeat one at a time. EM is run again for each new initial condition and the solution with the best likelihood is chosen. Trying initial conditions with fewer hyper-edited repeats balances the fact that expected counts tend to spread variation across repeats in early EM steps and allows us to settle on a set of highly confident predictions.

### Drosophila stocks

Drosophila strains were raised at a constant 25 °C, on standard molasses food, and under 12 h day/night cycles.

### Cloning and RNA editing analysis

To examine RNA-editing, total RNA was extracted from heads and thoraxes (20 per sample) of 1- to 2-day-old male Drosophila. RNA extractions were performed using TRIzol reagent (Invitrogen). Total RNA was transcribed into cDNA using M-MLV Reverse Transcriptase from Promega using an rdgA specific primer: RDGD-RT3 5 ^′^-GATTAATAGCATCGCACTCGAAGTAATCCC-3 ^′^. Edited cDNAs were amplified via PCR using target-specific primers: RDGINT-F2 5 ^′^-GTATGTATGTTTATCAACACCCTCC-3 ^′^ and RDGD-R3 5 ^′^-GACTTCATTCCAACGCTGTCGTTCTG-3 ^′^. The PCR product was purified using the Wizard®;SV Gel and PCR Clean-Up System from Promega (catalog number: A9282) from 1.5% agarose gel electrophoresis. Subsequently, 4 *μ*L of PCR product was cloned into One Shot®;TOP10 Chemically Competent E. coli cells using Zero Blunt®;TOPO®;PCR Cloning Kit from Invitrogen (catalog number: K2800J10), according to manufacturer’s guidelines. A total of 50 *μ*L solution containing the transformed cells were plated on kanamycin+agar plates. Plates were incubated overnight at 37 °C. Colonies picked from the plate were grown in kanamycin+ LB media overnight at 37 °C shaker at 200 RPM. DNA was isolated from 600 *μ*L of culture media using PureYield™Plasmid Miniprep System, according to manufacturer’s guidelines. Finally, 2 *μ*L of isolated DNA was used for the sequencing reaction using BigDye®;to obtain chromatograms for analysis.

### LoxP RNA 100BP paired-end sequencing

Total RNA, extracted by the above procedure, was sent to Genewiz for the preparation and deep sequencing of 100bp paired-end libraries. No polyA selection was preformed, but otherwise library prep and sequencing was done according to standard Genewiz methods.

### Generation of candidate alignments

The script used to process reads and generate candidate alignments can be found at https://github.com/wmckerrow/RepProfile/blob/master/utilities/make_candidate_alignments_genomic.sh. First, T’s in antisense reads are replaced with C. A’s in sense reads are replaced with G. Similarly, two masked genomic references are created by replacing A with G in one and T with C in the other. Two bam alignments are generated by using bwa aln (version 0.7.12) [47] to align masked reads to each of the masked references and subsequently merged into a single alignment. Reads for which some part of at least one of the read ends overlaps sequence labeled as the repeat of interest (FB4_DM, DNAREP1_DM or PROTOP/PROTOP_A/PROTOP_B) in the repeatmasker database (as downloaded from the UCSC table browser: genome.ucsc.edu, dm6 version) are extracted. Reads with mean base quality less than 30 were excluded. A repeat genome is generated from positions that are in or within 1kb of sequence labeled as the target repeat.

The repeat reads are aligned to the repeat genome, using the same masking procedure, this time retaining up to 10,000 secondary alignments with at most 4 mismatches (after masking.) The resulting combined bam file is sorted by read name and parsed by RepProfile.

### Simulation

The hypothetical repeat family was generated as follows: A random 1kb sequence was generated and copied 24 times – 12 in each orientation. For 10 copies in each direction, a 1kb of random flanking sequence was added to each end. The other four copies were paired to form two RNA duplexes. 1 kb of random sequence was added to each end of each duplex. For the other two simulations, the FB4_DM repeat reference was used.

In the first simulation, using the hypothetical repeat, both duplexes but none of the isolated repeats are simulated to be hyper-edited. One duplex is edited on the plus strand, and one on the minus strand. In the second simulation, using FB4_DM, only the cloned region was simulated to be hyper-edited. In the third simulation, again using FB4_DM, each of 13 editable FB4_DM had a 0.3 chance of being hyper-edited. This simulation was repeated 20 times and results were pooled. All the editable FB4_DM are in highly-expressed genes and greater than 1500 bases long. Hyper-editing is simulated in the direction of gene transcription.

In the first and third simulations (excluding the clone simulation), the simulated profile was generated as follows: Within each hyper-edited repeat, each editable position has a 0.5 chance of being edited. For each edited position, *p* is chosen uniformly between 0.001 and 0.997. To generate the profile at an edited position, the edited base G(C) is given probability *p*, the reference base A(T) is given probability 0.998−*p*, and the other two bases are given probability 0.001. Each position in any repeat has a 0.01 chance of being a SNP. For each SNP position, *p* is chosen uniformly between 0.001 and 0.997 and a non-reference base, *x*, is chosen uniformly at random. To generate the profile at SNP positions, base *x* is given probability *p*, the reference base is given probability 0.998−*p*, and the other two bases are given probability 0.001. For other positions, including all flanking sequence, the reference base has probability 0.997 and the other three bases have probability 0.001.

For the clone simulation, outside of the cloned region, the profile matched the reference genome, with each other base having probability 0.001 of appearing by simulated read error. Inside the cloned region, the profile was estimated from the clones.

For the hypothetical repeat family, half of the isolated repeats are transcribed in each direction. For the FB4_DM simulations, reads are drawn in proportion to exon coverage. FB4_DM not in genes are sampled at a low level.

For the FB4_DM simulation, 200,000 reads are drawn. For the hypothetical repeat simulation, 50,000 reads are drawn.

## Additional files

Additional file 1 Supplementary results. Descriptions of TE families with 1000 or more edit sites. (PDF 32 kb)

Additional file 2 Supplementary tables. List of all editing predictions in FB4_DM, DNAREP1_DM and PROTOP along with sequences for the rdgA clones. (XLSX 1,065 kb)

Additional file 3 rdgA FD4_DM. RNAstructure prediction for rdgA FD4_DM. The FD4_DM element forms a dsRNA structure that is too long to fit on a figure. (PDF 137 kb)

Additional file 4 Supplementary methods. Detailed description of RepProfile prior and simplifications for EM. Also included: rationale for choosing to focus on FB4_DM, DNAREP1_DM and PROTOP. (PDF 133 kb)

## Abbreviations

ADAR: Adenosine deaminase acting on RNA
ds: Double stranded
EER: Editing enriched region
EM: Expectation maximization
Hok: Hoppel killer
PPV: Positive predictive value
rdgA: retinal degeneration A
SMS: single-molecule sequencing (Helicos platform)
TE: Transposable element
