# Supplementary material for: Predicting RNA hyper-editing with a novel tool when unambiguous alignment is impossible
Source: BMC Genomics. 2017 Jul 10;18:522. doi: 10.1186/s12864-017-3898-9 (PMC5502491; doi:10.1186/s12864-017-3898-9)
Supplement: Supplementary file 1 — Supplementary results. Descriptions of TE families with 1000 or more edit sites. (PDF 32 kb) [file 12864_2017_3898_MOESM1_ESM.pdf]

SUPPLEMENTAL RESULTS

Here we include short descriptions RepProfile predictions for TEs with at least 1000 predicted editing sites. Summary of runtime and number of predictions for every TE can be found in the supplemental table, as can a full list of all predicted edit sites across all TEs.

BEL

RepProfile predicted 1086 edit sites in BEL repeats but did not make any highly confident predictions. Predicted edit sites are in the ranges chr2R:18575852-18581982 (GEFmeson intron) and chrX:3415072-3421199 (CG12535 intron).

Copia

RepProfile predicted 2084 edited sites in Copia repeats. However the only highly confident predictions were 62 edit sites in a region (chr3R:1698684-1699615) of a Myo81f intron. Other predictions were in intergenic and poorly assembled sequence.

DMCR1A

RepProfile predicted 2693 edit sites in DMCR1A. More than half of these are in two genomic regions that are highly confident predictions. 401 edit sites are predicted in chr3R:3450703-3456350, where there are DMCR1A elements on both strands. 1145 edit sites are predicted in chr2R:1306411-1333449, where there are also DMCR1A on both strands. Neither of these regions overlap annotated genes.

FW_DM

RepProfile predicted 2742 edit sites in FW_DM. More than 90% of these predictions are in three genomic regions. 1223 edit sites are predicted in chr3R:660095-666560 which includes two adjacent oppositely oriented FW_DM repeats inside a Myo81f intron. Another 716 sites are predicted in Myo81f at chr3R:1606987-1623276, which includes FW_DM elements both strands. 558 sites are predicted in an rl intron: chr2R:1097537-1101863. Of these three regions, only chr3R:1606987-1623276 is a highly confident prediction.

Gypsy

RepProfile predicted 3435 edit sites in Gypsy repeats. 13% are in the range spanning chr3R:3204734-3214018. This region is part of a Pzl intron and includes Gypsy repeats on both strands. 84% are in the region spanning chr2R:2808821-2839705 – part of a CG41378 intron that contains Gypsy repeats on both strands. Both regions include highly confident predictions.

ROO

RepProfile predicted 1316 edit sites in ROO. 96% of the predicted sites are in the genomic region chr3R:1596105-1626676 – a section of a Myo81f intron that contains ROO elements on both strands. This is a highly confident prediction.

ROVER

RepProfile predicted 1386 edit sites in ROVER. All edit sites are in the range chr2R:580416-591861, a section of a CG45781 intron that contains ROVER elements on both strands. This is a highly confident prediction.

S_DM

RepProfile predicted 1332 edit sites in S_DM. Like FB4_DM, S_DM elements are capable of forming dsRNA by self-complementarity. Highly confident hyper editing predictions are made in S_DM elements at the following 10 locations:

Position	Gene
chr3R:4683231-4684935	CG43427
chr3R:611446-613180	Myo81f
chr3R:1343650-1345383	Myo81f
chr3R:1642493-1642860	Myo81f
chr2R:2194058-2195781	CG17684
chr4:420027-421232	intergenic
chr3L:25608432-25610027	CG45782
chr3L:25414448-25416178	CG45782
chr3L:27280020-27281201	Dbp80
chr3L:27387332-27389063	Dbp80
