# Supplementary figures and images for: Predicting RNA hyper-editing with a novel tool when unambiguous alignment is impossible
Source: BMC Genomics. 2017 Jul 10;18:522. doi: 10.1186/s12864-017-3898-9 (PMC5502491; doi:10.1186/s12864-017-3898-9)

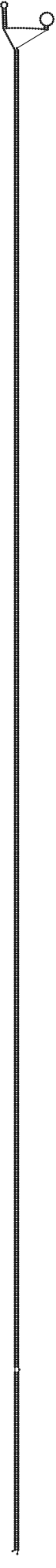

Supplement: Supplementary file 3 — rdgA FD4_DM. RNAstructure prediction for rdgA FD4_DM. The FD4_DM element forms a dsRNA structure that is too long to fit on a figure. (PDF 137 kb) [file 12864_2017_3898_MOESM3_ESM.pdf]
