# Supplementary material for: Predicting RNA hyper-editing with a novel tool when unambiguous alignment is impossible
Source: BMC Genomics. 2017 Jul 10;18:522. doi: 10.1186/s12864-017-3898-9 (PMC5502491; doi:10.1186/s12864-017-3898-9)
Supplement: Supplementary file 4 — Supplementary methods. Detailed description of RepProfile prior and simplifications for EM. Also included: rationale for choosing to focus on FB4_DM, DNAREP1_DM and PROTOP. (PDF 133 kb) [file 12864_2017_3898_MOESM4_ESM.pdf]

SUPPLEMENTAL METHODS

The Prior

The specific RepProfile prior used in our analysis is defined follows. The three repeat states are hyper_f (hyper edited on the + strand), hyper_r (hyper edited on the - strand), and not (not hyper edited). Each repeat state is an independent draw from the follow distribution:

$$\begin{aligned}
P(H_k = \text{hyper_f}) &= 0.01 \\
P(H_k = \text{hyper_r}) &= 0.01 \\
P(H_k = \text{not}) &= 0.98
\end{aligned}$$

The position states are ref (not SNP or edit), edit_f (edited on the forward strand), edit_r (edited on the reverse strand), and SNPx (SNP base ref-x (mod 4) where A=0,C=1,G=2,T=3, possibly unfixed.)

$$\begin{aligned}
P(T_i = \text{ref} | H_k(i) = \text{hyper_f}, ref = A) &= 0.495 \\
P(T_i = \text{ref} | H_k(i) = \text{hyper_f}, ref \neq A) &= 0.99 \\
P(T_i = \text{ref} | H_k(i) = \text{hyper_r}, ref = T) &= 0.495 \\
P(T_i = \text{ref} | H_k(i) = \text{hyper_r}, ref \neq T) &= 0.99 \\
P(T_i = \text{ref} | H_k(i) = \text{not}) &= 0.99 \\
P(T_i = \text{edit_f} | H_k(i) = \text{hyper_f}, ref = A) &= 0.495 \\
P(T_i = \text{edit_r} | H_k(i) = \text{hyper_r}, ref = T) &= 0.495 \\
P(T_i = \text{SNP1}) = P(T_i = \text{SNP3}) &= 0.0025 \\
P(T_i = \text{SNP2}) &= 0.005
\end{aligned}$$

The genome profile G is drawn from one of the following Dirichlet distributions, where the dth dimension corresponds to nucleotide $ref - d$ (mod 4).

$$\begin{aligned}
G_i | T_i = \text{ref} &\sim Dir(10.0, 0.01, 0.01, 0.01) \\
G_i | T_i = \text{edit_f} &\sim Dir(1.0, 0.01, 1.0, 0.01) \\
G_i | T_i = \text{edit_r} &\sim Dir(1.0, 0.01, 1.0, 0.01) \\
G_i | T_i = \text{SNP1} &\sim Dir(1.0, 1.0, 0.01, 0.01) \\
G_i | T_i = \text{SNP2} &\sim Dir(1.0, 0.01, 1.0, 0.01) \\
G_i | T_i = \text{SNP3} &\sim Dir(1.0, 0.01, 0.01, 1.0)
\end{aligned}$$

Probability Calculations for EM

Glossary of Random Variables:

- $R = R_1, \dots, R_m$ is the set of **R**ead sequences
- $A = A_1, \dots, A_m$ is the **A**lignment of each read
- $X = X_1, \dots, X_r$ is the relative e**X**pression (coverage) of each TE
- $G = G_1, \dots, G_n$ is the **G**enome profile (probability of A/C/G/T sequenced) at each TE position
- $H = H_1, \dots, H_r$ are the **H**yper parameters, representing underlying sources of variation in G such as hyper editing.
- $T = T_1, \dots, T_n$ are the variation **T**ypes of each position such as SNPs or editing targets.
- $U(A, R) = U_1, \dots, U_n$ are the number of A, C, G, T aligned at each position.
- $V(A) = V_1, \dots, V_r$ are the number of reads aligned to each repeat.

In this application, the alignment, A, is treated as a hidden variable, the read set, R, is known, and X, H, T, and G parameters to be estimated. Thus performing an EM update requires computing the following:

$$X^{(t+1)}, H^{(t+1)}, T^{(t+1)}, G^{(t+1)} = \underset{X,H,T,G}{\arg\max}\, \mathbb{E}[\log P(X,H,T,G,A,R)|R, X^{(t)}, H^{(t)}, T^{(t)}, G^{(t)}] \quad (1)$$

where the parenthetical superscript (t) refers to the EM estimate at step t. Following the directed graph in figure 10, the joint distribution factors as follows:

$$P(X,H,T,G,A,R) = P(X)P(A|X)P(H)P(T|H)P(G|T)P(R|A,G) \quad (2)$$

where

- $P(X) \propto 1$.
- $P(A|X) = \prod_j h(A_j)\frac{X_{A_j}}{\ell_{A_j}}$, where ℓ_j is the length of repeat j and $h(A_j)$ is probability of the indels in A_j.
- $P(G|T) = \prod_i \frac{1}{B(\alpha_{T_i})} \prod_{x \in \{a,c,g,t\}} (G_i^x)^{\alpha_{T_i}^x - 1}$, where $\alpha_{T_i}^x$ is the Dirichlet constant for base x in state T_i.
- $P(R|A,G) = \prod_i \prod_{x \in \{a,c,g,t\}} (G_i^x)^{U_i^x(A,R)}$.

Thus letting $\mathbb{E}_A$ refer to expectation conditioned on $R, X^{(t)}, H^{(t)}, T^{(t)}, G^{(t)}$,

$$X^{(t+1)}, H^{(t+1)}, T^{(t+1)}, G^{(t+1)} = \underset{X,H,T,G}{\arg\max}\, \mathbb{E}_A[\log P(X,H,T,G,A,R)] \quad (3)$$

$$= \underset{X,H,T,G}{\arg\max}\, \mathbb{E}_A[\log[P(X)P(A|X)P(H)P(T|H)P(G|T)P(R|A,G)]] \quad (4)$$

$$= \underset{X}{\arg\max}\, \mathbb{E}_A[\log P(A|X)], \quad (5)$$

$$\underset{H,T,G}{\arg\max} \log P(H,T,G) + \mathbb{E}_A[\log P(R|A,G)] \quad (6)$$

For term **??**,

$$\arg\max_X \mathbb{E}_A[\log P(A|X)] = \arg\max_X \sum_k \mathbb{E}_A[V_k(A)] \log X_k + \text{terms not depending on } X \tag{7}$$

$$= \frac{V(A)}{\sum V(A)} \tag{8}$$

And for term **??**, considering each repeat separately,

$$\max_{H,T,G} \ldots = \max_{H,T,G} \log P(H,T,G) + \mathbb{E}_A[U] \cdot \log G \tag{9}$$

$$= \max_{H,T} \log P(H,T) - \sum \log B(\alpha_T) + \max_G (\mathbb{E}_A[U] + \alpha_T - 1) \cdot \log G \tag{10}$$

We can then let

$$\hat{G}_T = \arg\max_G (\mathbb{E}_A[U] + \alpha_T - 1) \cdot \log G = \frac{\mathbb{E}_A[U] + \alpha_T - 1}{\sum \mathbb{E}_A[U] + \alpha_T - 1} \tag{11}$$

and

$$P_{\hat{G}_T}(i) = (\mathbb{E}_A[U_i] + \alpha_{T_i} - 1) \log \hat{G}_{T_i} \tag{12}$$

yielding

$$\max_{H,T,G} \ldots = \max_H \log P(H) + \sum_i \max_{T_i} \left(\log P(T_i|H) - \log B(\alpha_{T_i}) + P_{\hat{G}_T}(i) \right) \tag{13}$$

As there are only a small number of repeat states and a small number of position states, we can calculate

$$\hat{T}_H = \arg\max_T \left(\log P(T|H) - \log B(\alpha_T) + P_{\hat{G}_T} \right) \tag{14}$$

for each H by brute force and let

$$P_{\hat{T}_H} = \log P(\hat{T}_H|H) - \log B(\alpha_{\hat{T}_H}) + P_{\hat{G}_{\hat{T}_H}} \tag{15}$$

Finally we can calculate

$$H^{(t+1)} = \max_H P(H) + P_{\hat{T}_H} \tag{16}$$

the max for H at step $t+1$. We can then plug $H^{(t+1)}$ to get the max for T and G: $T^{(t+1)} = \hat{T}_{H^{(t+1)}}$ and $G^{(t)} = \hat{G}_{T^{(t+1)}}$. We force all elements of $G^{(t)}$ to be ≥ 0.001 to prevent the probability of any candidate alignment from becoming 0 or worse – all the candidate alignment for a particular read.

$\mathbb{E}_A[U]$ and $\mathbb{E}_A[V]$ can then be approximated by calculating the joint probability of each read sequence for each candidate alignment under the profile $G^{(t)}$ with expression levels $X^{(t)}$:

$$\mathbb{E}_A[U] = \sum_j \sum_{A_j} U(A_j, R_j) P(A_j | G^{(t)}, X^{(t)}, R_j) \tag{17}$$

$$= \sum_j \sum_{A_j} U(A_j, R_j) \frac{\prod G^{(t)}(U(A_j, R_j))}{\sum_{A'_j} \prod G^{(t)}(U(A'_j, R_j))} \tag{18}$$

and

$$\mathbb{E}_A[V] = \sum_j \sum_{A_j} V(A_j) P(A_j | G^{(t)}, X^{(t)}, R_j) \tag{19}$$

$$= \sum_j \sum_{A_j} V(A_j) \frac{\prod G^{(t)}(U(A_j, R_j))}{\sum_{A'_j} \prod G^{(t)}(U(A'_j, R_j))}. \tag{20}$$
